# Designing strategies of small-molecule compounds for modulating non-coding RNAs in cancer therapy

**DOI:** 10.1186/s13045-022-01230-6

**Published:** 2022-02-05

**Authors:** Rongyan Zhao, Jiahui Fu, Lingjuan Zhu, Yi Chen, Bo Liu

**Affiliations:** 1 State Key Laboratory of Biotherapy and Cancer Center, and Department of Gastrointestinal Surgery, West China Hospital, Sichuan University, Chengdu, 610041 China; 2 School of Traditional Chinese Materia Medica, Key Laboratory of Structure-Based Drug Design and Discovery of Ministry of Education, Shenyang Pharmaceutical University, Shenyang, 110016 China

**Keywords:** Non-coding RNA (ncRNA), Cancer therapy, Designing strategy, Small-molecule compound, Drug discovery

## Abstract

Non-coding RNAs (ncRNAs) have been defined as a class of RNA molecules transcribed from the genome but not encoding proteins, such as microRNAs, long non-coding RNAs, Circular RNAs, and Piwi-interacting RNAs. Accumulating evidence has recently been revealing that ncRNAs become potential druggable targets for regulation of several small-molecule compounds, based on their complex spatial structures and biological functions in cancer therapy. Thus, in this review, we focus on summarizing some new emerging designing strategies, such as high-throughput screening approach, small-molecule microarray approach, structure-based designing approach, phenotypic screening approach, fragment-based designing approach, and pharmacological validation approach. Based on the above-mentioned approaches, a series of representative small-molecule compounds, including Bisphenol-A, Mitoxantrone and Enoxacin have been demonstrated to modulate or selectively target ncRNAs in different types of human cancers. Collectively, these inspiring findings would provide a clue on developing more novel avenues for pharmacological modulations of ncRNAs with small-molecule drugs for future cancer therapeutics.

## Introduction

Non-coding RNAs (ncRNAs) is well-known as a class of RNA molecules that do not encode proteins, including microRNAs (miRNAs), long non-coding RNAs (lncRNAs), Circular RNAs (circRNAs), Piwi-interacting RNAs (piRNAs), ribosomal RNAs (rRNAs), and transfer RNAs (tRNAs) with known biological functions, as well as those with unknown functions [1]. And, ncRNAs have been widely reported to lack clear potential to encode proteins or functional peptides, but regulate the expression of other genes [2, 3]. Notably, ncRNAs are closely involved in many human diseases [4–6], especially cancer, because they act their essential roles in several important biological processes, including gene expression, epigenetic regulation, and autophagy [7–10].

Hitherto, it has been generally accepted that proteins are the targets of most drugs in human diseases [11–13]. After all, protein is the material basis of life, and almost all life activities are closely related to proteins. The strategy of targeting pathogenic proteins has brought many new drugs in the past decade [14, 15]. However, starting from the blueprint of the human genome, with the in-depth understanding of genomics, proteomics and other biological molecules, the limitations of targeting proteins have begun to be realized [16]. First, only 2% of human DNA encodes proteins. In addition, 10–15% of the approximately 20,000 expressed human proteins are considered to be disease-related, that is, 0.2% of the genome codes for disease-related proteins [17, 18]. Secondly, targeting proteins with small molecules requires the identification of specific binding sites on the spatial structure of proteins, which is challenging because most proteins are “undruggable” [19].

Of note, ncRNAs may account for 70% of the whole human genome [20], making the modulation of ncRNAs as a therapeutic strategy more attractive. Moreover, because ncRNAs are located upstream of proteins, they may be able to target such undruggable proteins at an early stage. For example, ribocil binds to the ncRNA structural unit of riboflavin, FMN riboswitch, and inhibits it cellular production and metabolites [21]. Designing structure-specific ligands is reported to target a common structure in the Dicer processing sites of miRNAs cluster and thereby inhibiting their biogenesis [22]. Using high-throughput screening, the small-molecule compound AC1NOD4Q (ADQ) has been identified as a leading candidate compound for targeting lncRNAs, and ultimately inhibiting cancer cell metastasis via the Wnt/β-Catenin signaling pathway [23]. In addition, some candidate small-molecule drugs targeting miRNAs have entered clinical trials for cancer treatment [24, 25].

With the emergence of ncRNAs and innovations in high-throughput screening technology, the mechanisms of ncRNAs in cancer is being further explored. Currently, the number of small molecules for regulating ncRNAs have been increasing. Therefore, we attempt to elucidate the reasons why ncRNAs are modulated by small-molecule compounds, and highlights the designing strategies for discovery of small-molecule compounds for modulating ncRNAs in cancer, which may provide a new clue on exploiting potential small-molecule drugs.

## Non-coding RNAs as emerging druggable targets in cancer

Non-coding RNAs (ncRNAs) can form different secondary structures through the typical Watson–Crick base pairing principle and atypical bond-mediated interactions, including helices, hairpins, loops, bumps, and pseudoknots. The further interaction of these structural elements to form more complex three-dimensional structures is expected to lead to enough drug conformations for small-molecule binding and recognition. For example, Linezolid and Ribocil are excellent small-molecule compounds, which are in line with the classic drug Lipinski’s rule of five, have small polar total surface area, and have good cell membrane permeability and no apparent toxicity. Crucially, they perform their pharmacological effects by binding to structural “pockets” of RNA targets, unlike most small-molecule drugs that target disease-causing proteins. Due to the simple composition of ncRNAs, compared with the complex spatial structure of proteins, small-molecule compounds can easily enter the key sites that affect their functions [19]. Additionally, the diversity of ncRNA folding space makes up the disadvantage of having only four nucleotides, and also provides an opportunity for small molecular compounds to target ncRNAs [26]. ncRNAs are involved in intracellular protein assembly, and diseases with “no cure” may be improved at the RNA level before protein production [27, 28].

The therapeutic use of ncRNAs has been drawn a rising attention since they were first discovered, especially in the last decade, ncRNAs such as miRNA and lncRNA have become the focus of research [29–32]. ncRNAs, which were initially ignored as transcriptional noise or artefactual transcripts, have their complex biological functions. They play the key roles in carcinogenesis or tumor inhibition, and are also regarded as crucial biomarkers for cancer diagnosis and prognosis. For example, overexpressed miR-122 has been reported to reduce Hes1 and NOTCH1 in A549 stem cells, and by blocking the NOTCH1 pathway, miR-122 can reduce the resistance of A549 stem cells to gefitinib, and thereby suppressing cancer cell proliferation and migration [33]. Moreover, miR-34a has been shown to have an anti-tumor activity in breast cancer, which blocks the NOTCH1 pathway by functionally targeting *Notch1*, thereby regulating cell proliferation, migration, and invasion [34].

Of note, lncRNAs have a transcript length of more than 200 nucleotides [35], which participated in a variety of cellular processes, including chromatin structure regulation, transcriptional regulation, reinforcement or suppression of protein activity, regulation of nuclear bodies and play important roles in cancer, including epigenetic regulation, interactions with miRNAs, cell cycle regulation, and mediating hormone-induced cancer [36, 37]. DANCR has been found to the 3 'untranslated region of *CTNNB1* mRNA and block miRNA-mediated *CTNNB1* translation inhibition by competitive binding to miR-214 and other sites, resulting in increased *CTNNB1* protein expression and activation of the Wnt signaling pathway [38]. Myocardial infarction-associated transcript (MIAT), another lncRNA, has been shown to promote gastric cancer growth and metastasis by regulating the miR-141/DEAD-box RNA helicase 5 (DDX 5) pathway [39]. Further, The HOX transcript antisense intergenic RNA (HOTAIR), a well-known lncRNA, was shown to HOTAIR deletion can inhibit the proliferation, migration, invasion and PI3K/ATK signaling pathway of gastric cancer cells [40].

**Fig. 1:**
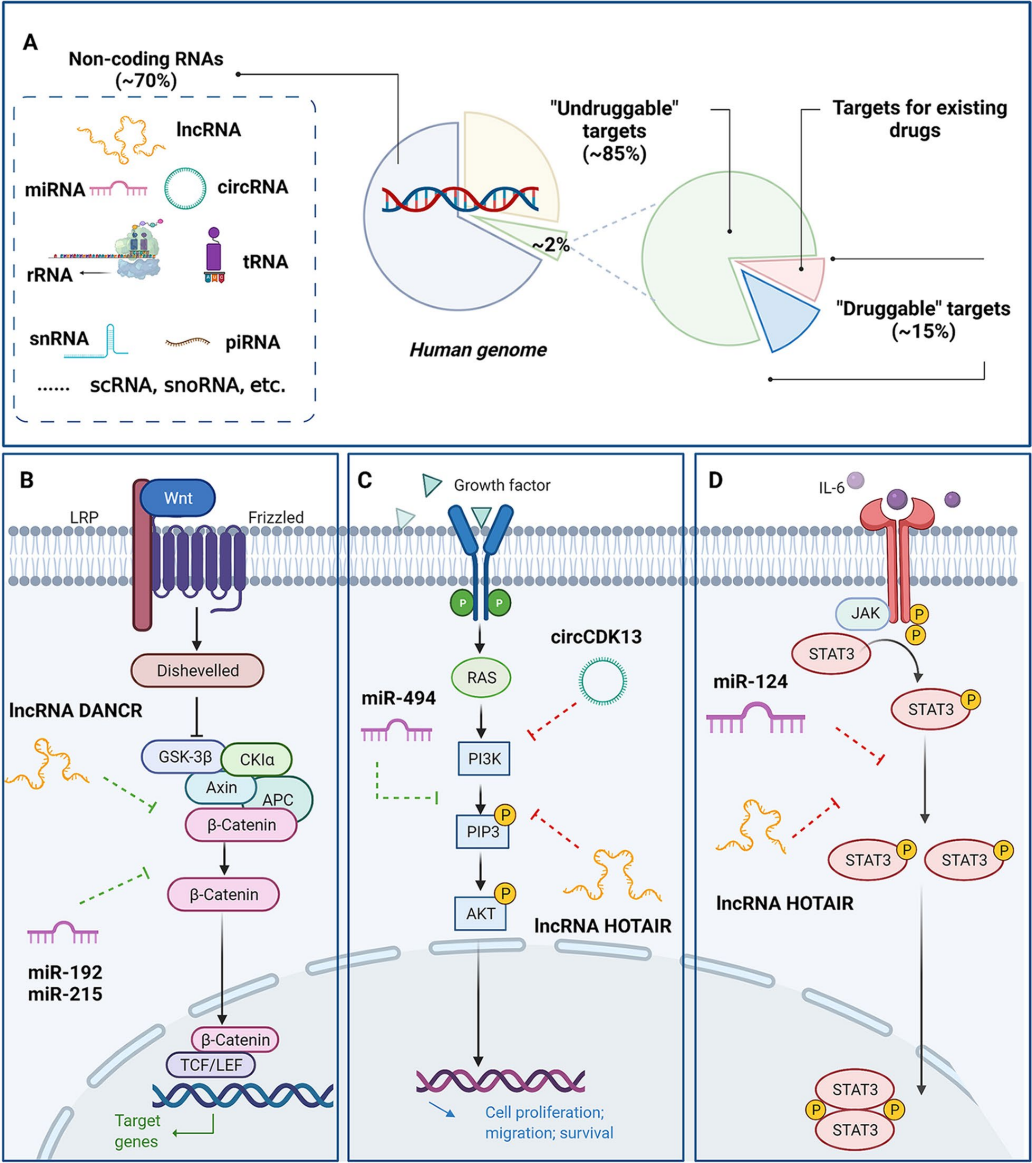
ncRNA as potential therapeutic target for cancer. **A** Abundance of non-coding RNA species: as the pie chart shows, only about 2% of the human genome is used to encode proteins (~ 20,000 proteins). As illustrated at the right, among about 20,000 proteins that make up the human proteome, only 15% are traditionally druggable protein targets. In contrast, ncRNAs, such as lncRNAs, miRNAs and tRNAs, occupy ~ 70% of the genome. Thus, ncRNAs may be a source of potential targets. **B** Wnt/β-catenin pathway, **C** PI3K/AKT pathway, **D** JAK/STAT pathway. Notably, these above-mentioned signaling pathways involved in ncRNAs in the development of cancer are not limited to the above three kinds and the red dotted line represents inhibition, and the green dotted frontal line represents activation

CircRNAs, which are a class of ncRNA molecules, are characterized by the absence of a 5' terminal cap and a 3' terminal poly (A) tail which form a ring structure with covalent bonds [41]. Compared with miRNAs and lncRNAs mentioned above, there are fewer studies on circRNAs, but this does not affect the key role of its in cancer. For example, circCDK13 has been reported to be a novel circRNA that inhibits the progression of hepatocellular carcinoma, which are possibly mediated via the PI3K/AKT and JAK/STAT pathways [42]. Hsa_circ_0000515 is a novel circRNAs participating in breast cancer by regulating the miRNA-296-5p/CXCL10 axis [43]. Circ YAP1 can act as a tumor suppressor in gastric cancer cells by targeting miR-367-5p/p27Kip1 axis [44]. Together, these findings suggest that circular RNA is a potential target for future diagnosis and treatment [45] (Fig. 1).

In addition to the common non-coding RNAs mentioned above, others, such as tRNA, piwi-intervening RNAs (piRNAs), are also biologically important in cancer. For instance, tRNA-derived fragments (TRFs) and tRNA half fragments (tiRNAs) are small ncRNAs derived from precursor tRNA or mature tRNA. TRFs and tiRNAs have been shown to regulate the progression of various cancers, including lung cancer, colorectal cancer, prostate cancer, etc. [46]. PiRNA-36712 has been found to suppress the malignant phenotype of breast cancer cells by interaction with the SEPW1 pseudogene SEPW1P RNA [47].

Moreover, ncRNAs have a spatial configuration to be directly targeted, and complex biological functions support the regulation of ncRNAs by small-molecule compounds [35, 48]. Inspiringly, a small-molecule compound that binds to human ribosomes has been found, and three kinase and topoisomerase inhibitors have been screened through an “AbsorbArray” [49, 50]. Hence, ncRNAs are regarded as the most attractive potential cancer therapeutic targets.

## Designing strategies of small-molecule compounds for modulating ncRNAs in cancer

From the initial random discovery to intelligent screening, the increasing number of designing strategies has been being used to discover more small-molecule compounds for regulating ncRNAs in cancer therapy. Herein, we focus on summarizing the following methods, including high-throughput screening approach [51–53], small-molecule microarray (SMM) approach [54], structure-based designing approach [55] and other approaches.

### High-throughput screening approach

Of note, high-throughput screening, which is associated with combinatorial chemistry, is an experimental method based on molecular and cellular level and using microporous plates as carriers for simultaneous detection of multiple samples. Considering the sensitivity, rapidity, efficiency and automaticity of high-throughput screening and the large amount of ncRNAs and small-molecular compounds, this technology is more suitable for the rapid screening of small-molecular compounds that regulate ncRNAs. Notably, screening models are an important part of the overall high-throughput screening process, including molecular and cellular drug screening models [56]. How to determine the model, you can refer to the following suggestions: Molecular level drug screening model is the most used model in high-throughput screening. According to the types of biomolecules, drug screening models at the molecular level are mainly divided into receptor, enzyme, channel, gene, and other types of models. These models have the characteristics of specific drug action targets, and information of drug action mechanism can be obtained directly by using these models [57, 58]. Previous studies established a high-throughput screening method for inhibitors targeting the structure of the 16S rRNA coding region and the HIV-RRE RNA structure to search for new compounds similar to aminoglycoside antibiotics with higher affinity or acting on other sites of the same nucleic acid, as well as new compounds that are not easily metabolized and inactivated. This approach can be based on the principle that the fluorescence of pyrene is quenched when amino analogs containing pyrene are bound to RNA [59]. Cell-level drug screening model is designed to observe the effect of screened samples on cells, but it cannot reflect the specific pathway and target of drug action, and can only reflect the comprehensive effect of drugs on cell growth and other processes. The most important model for cell-level drug screening is reporter gene analysis because transcription factors and gene expression-related factors are important targets for drug action. Interestingly, reporter gene assay (a cell-level drug screening model) is often used to obtain small-molecule compounds that regulate ncRNAs through high-throughput screening techniques. For example, the miR-21 complementary binding site inserted downstream of the firefly luciferase gene at 3'UTR causes endogenous expression of the reporter gene to miR-21, resulting in decreased luminescence. On the contrary, functionally inhibited miR-21 can relieve the translation inhibition of luciferase and enhance fluorescence intensity [60]. In this high-throughput screening of more than 300,000 small molecules, a new class of acetamide miR-21 inhibitors has been found. Through the structure–activity relationship study, a series of compounds was optimized based on compound 1 (Table 1, **1**), and it was found that compound **12** (Table 1, **2**) could inhibit the transcription of miR-21, resulting in a significant decrease in both primary and mature levels of miR-21. In addition, compound **12** could inhibit cervical cancer cells, increase the expression of caspase-3/7, and thus inducing Hela cell apoptosis, which suggests that compound 12 might have a potential as a promising therapeutic agent for miR-21-related diseases.

**Table 1:** Small-molecule compounds based on high-throughput screening approach

Compound	Name	Target	Structure	Cancer type	References
**1**	Compound **1**	MiR-21		Cervical cancer	[60]
**2**	Compound **12**				
**3**	Enoxacin	siRNA, MiRNA		Breast cancer, cervical cancer, Prostate cancer	[61]
**4**	Small molecule **1**	MiR-122		HCV, liver cancer	[62]
**5**	WG-1	FMN riboswitches		Lung adenocarcinoma, leukemia, antibacterial	[69]
**6**	WG-3				

To discover small-molecule compounds that regulate ncRNAs, San and Connelly used GFP and luciferase as reporter genes to design complementary sequences containing miRNA or siRNA in their 3ʹ-UTR, respectively. These reporter genes were then transferred into the cell line by stable or transient transfection. In cells, miRNA, or small interfering RNA (siRNA) can inhibit the expression of these reporter genes in a sequence-specific manner. Enoxacin (Table 1, **3**) was identified as a small-molecule compound that can enhance siRNA-mediated mRNA degradation and thereby promoting endogenous miRNA biosynthesis [61]. Additionally, small molecule **1** (Table 1, **4**) has been demonstrated to be a new inhibitor of miR-122 through high-throughput screening [62]. The reporter gene psiCHECK-miR122 was constructed by inserting the complementary sequence of mature miR-122 into the downstream of *Renilla* luciferase gene, and stable Huh7 reporter cell line was generated. When miR-122 binds to the corresponding target sequence, *Renilla* luciferase expression declines. The steps for screening and identifying miR-122 inhibitors are as follows: (1) primary small-molecule screening in psiCHECK-miR122 assay; (2) initial hit compounds identified by dose–response assay; (3) secondary assay using psiCHECK-control reporter to exclude nonspecific luciferase activators; (4) secondary assay using miR-21 luciferase reporter to exclude generic miRNA inhibitors; (5) qRT-PCR assay to measure intracellular levels of miR-122 and other miRNAs. Interestingly, high-throughput screening techniques can be used to identify small-molecule inhibitors of any miRNA of an interest once the appropriate report analysis has been constructed [62].

After decades of development, high-throughput screening technology is relatively mature in the field of drug research and development [63], and is used to identify and regulate small ncRNA molecules, also including methods based on the mass spectrometry and fluorescence energy resonance transfer [64, 65]. Mass spectrometry (MS) is commonly used to obtain molecular structure information by measuring ion charge mass ratios (charge mass ratio) [66]. With the successive progress of matrix-assisted laser desorption ionization (MALDI) and electrospray ionization (ESI) technologies, MS has been widely applied. Notably, MS can even be used as a screening tool. For example, the interaction between a series of aminoglycoside antibiotics and two strongly related RNA structures corresponding to rRNA decoding sites can be elucidated by Fourier transform ion cyclotron resonance (FT-ICR) mass spectrometry [67]. One of the earliest applications of ESI–MS was in the identification of 2-aminobenzimidazole, which specifically binds to the IIA subdomain of the internal ribosomal entry site of the hepatitis C virus, resulting in reduced HCV-RNA replication [68]. Moreover, affinity selection mass spectrometry (AS-MS) is also suitable for high-throughput screening of small molecules that bind to ncRNAs. The automatic ligand recognition system (ALIS), which is a label-free AS-MS platform, was applied for high-throughput screening of small molecules as large combinatorial mixtures and for testing their binding to target macromolecules [69]. As an “indirect” AS-MS technique, ALIS uses size exclusion chromatography to isolate target ligand complexes from unconjugated species, then dissociates ligands from the complexes under denaturation conditions, and identifies the previously bound ligands by MS. In this manner, the specific binding of five natural ligands to their respective RNA riboswitches was tested using the ALIS platform. The natural and synthetic ligands binding to FMN ribose switches were further characterized in ALIS. In high-throughput screening of FMN riboswitches using 53,000 antimicrobial compounds, the binding of ribocil was successfully detected by ALIS, which was initially determined by phenotypic screening. In addition, compounds WG-1 (Table 1, **5**) and WG-3 (Table 1, **6**) with high affinity and competitive binding to FMN riboswitches were found.

High-throughput screening technology organically combines advanced technologies such as chemistry, genomic research, biological information, and automated instruments into a new model that has the characteristics of fast and efficient, so that the cost and time of research will be reduced. More recently, although considerable progress has been achieved in its research and application, there are still many problems, such as the evaluation criteria for high-throughput screening models and the difficulty of handling some complex target screening.

### Small-molecule microarray approach (SMM)

SMM, a powerful technology, can evaluate the binding ability of biomolecules to large compound libraries, and it is also an effective way to screen small-molecule compounds regulating ncRNAs [70, 71]. In simple terms, SMM depends on small molecules being printed on glass surface of that thousands of spots are deposited in the form of arrays, and then screened by fluorescent-labeled biomacromolecules. Through deconvolution of highly enriched points of target molecules, the binding ability of the small molecules with target biomolecules can be identified and evaluated [72].

**Fig. 2:**
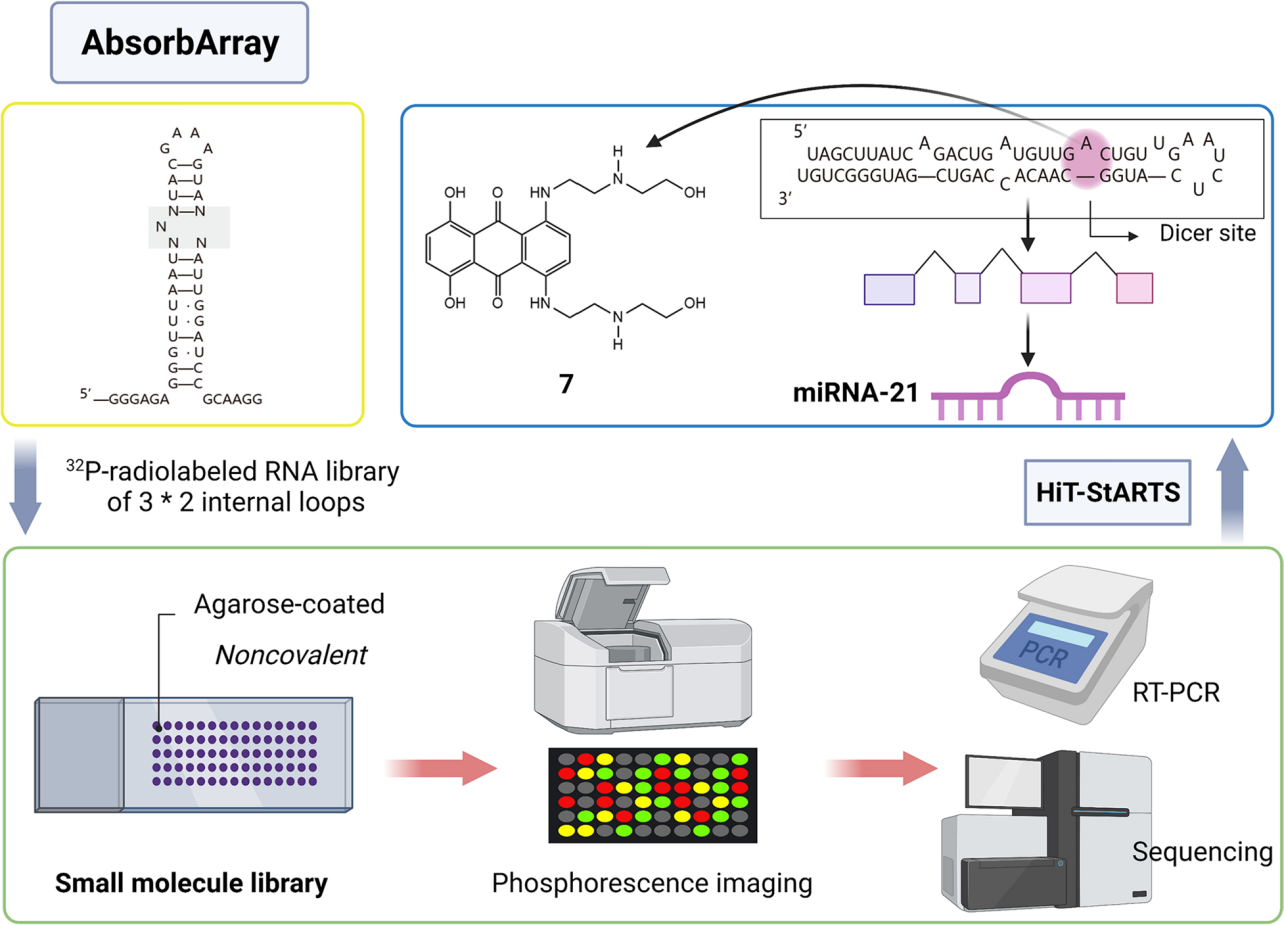
“AbsorbArray” is reported to be an approach of SMM. The small-molecule library is attached to the agarose coated microarray surface in a non-covalent manner. It is then incubated with ^32^P radiolabeled RNA Library of 3 × 2 internal loops and washed. Later, phosphorescence imaging is carried out. Finally, the radioactive spots are isolated and **7** obtained by RT-PCR and sequencing

Previously, large-scale SMM screening was used to identify different protein targets of small molecular ligands [73, 74]. Similarly, this method can also be used to find small-molecule compounds targeting RNA and ncRNAs [75]. For example, small-molecule microarrays are very valuable for identifying compounds that bind to biological molecules, but the strategy of immobilizing compounds by adding functional groups that allow covalent bonding is not perfect [76]. This modification may affect the molecular recognition of the natural target of the parent compound. However, AbsorbArray allows small molecules to adhere to the surface in a non-covalent manner and is used to detect whether the compound binds to the motif in ncRNA. Excitingly, mitoxantrone, a topoisomerase inhibitor, was discovered based on this method. In MDA-MB-231 cells, mitoxantrone binds to the A bulge at the Dicer site of pre-miR-21, which inhibits Dicer processing. This resulted in decreased levels of mature miR-21, while the level of pre-miRNA-21 was increased, and the aggressive phenotype triggered by the high expression of miR-21 was reversed in TNBC cells [50] (Fig. 2).

**Fig. 3:**
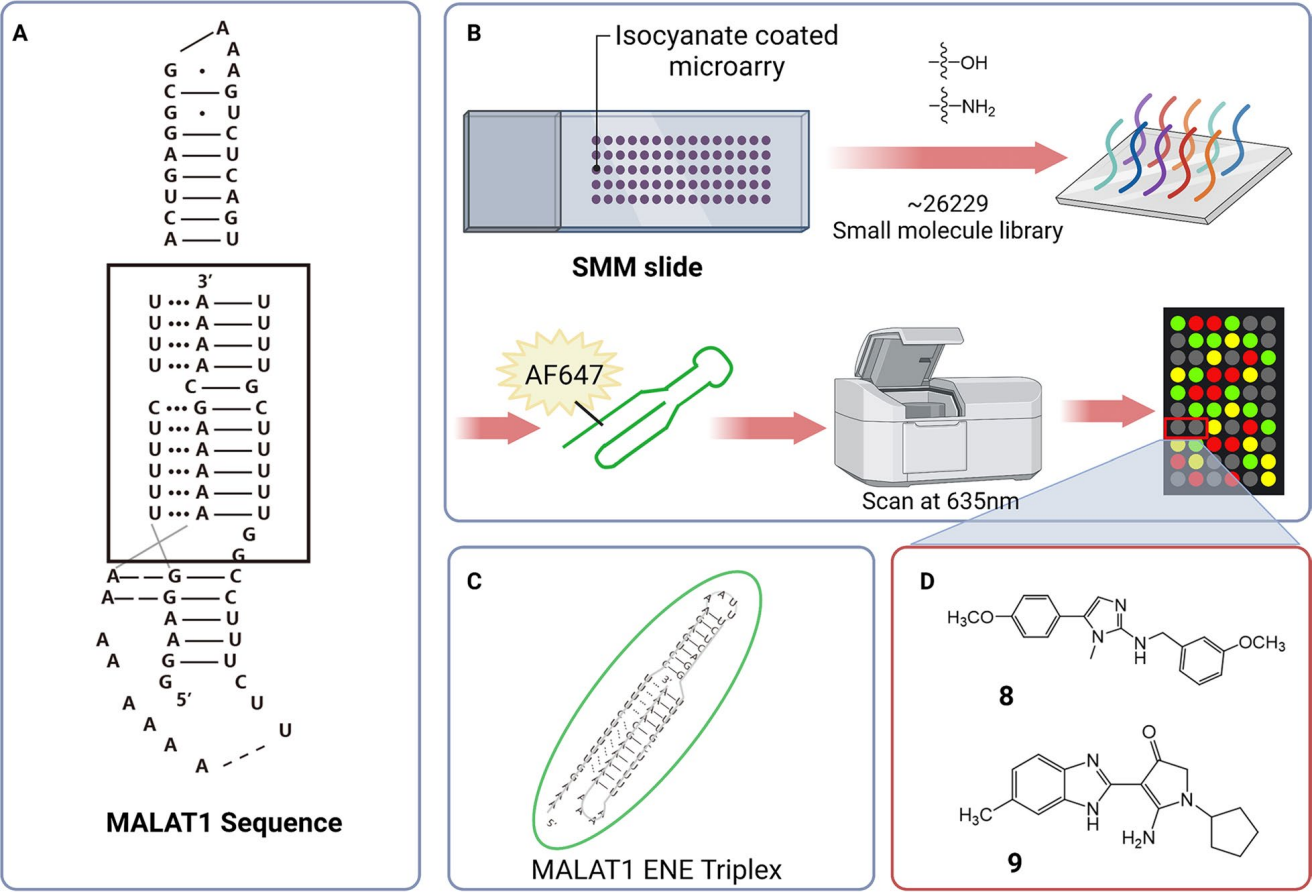
Small-molecule compounds for regulating MALAT1 based on SMM. (**A**) MALAT1 sequence, within which the ENE can be boxed. (**B**) SMM screening diagram. Small molecules were covalently attached to isocyanate-coated glass slides by chemical methods. The fluorescent labeled MALAT1 triple helix was incubated with the slide. Then the slides were washed to remove the unbound oligonucleotides, and fluorescence scanning imaging was performed at 635 nm. (**C**) MALAT1 element for nuclear expression (ENE) triplex. (**D**) The structures of compound **8** and **9**

Metastasis-associated lung adenocarcinoma transcript 1 (MALAT1), originally identified as a prognostic biomarker of advanced metastatic non-small cell lung cancer, is a highly enriched and ultra-conserved nuclear lncRNA. Subsequently, MALAT1 has been found to promote the proliferation of breast cancer cells by activating the PI3K/Akt/mTOR and Wnt/β-catenin pathways [77]. In addition, since the same miR-1 binding element present in the 3'-UTR of MALAT1 and cell division cycle 42(CDC42), MALAT1 can act as a competitive endogenous lncRNA to bind with miR-1, activate the expression of CDC42, and induce the migration and invasion of breast cancer cells [78]. Downregulation of MALAT1 using shRNAs in MCF7 cells resulted in inhibition of breast cancer cell-mediated angiogenesis in vitro, as well as cell proliferation and migration [79]. MALAT1, a lncRNA, is not only associated with breast cancer, but influences osteosarcoma progression by modulating cyclin-dependent kinase 9 (CDK9) expression via sponging miR-206 [80]. Structurally, MALAT1 has been shown to have a unique triple helix [81]. In summary, lncRNA MALAT1 is well-prepared for the unique regulation of the biomolecules by small-molecular compounds. The team of Donlic, A and Hargrove, AE brought exciting news to this idea with the discovery of the first selective ligand targeting the triple helix of MALAT1 [82]. Later, investigator used a small-molecule microarray method to perform a new round of screen (isocyanate-coated glass slides and about 26,229 commercially-available derivatives of amino- or hydroxyl-functional groups reacted to obtain a library of small molecules immobilized on the chip, fluorescently labeled to target the triple helix region of MALAT1, and the spots showing highly fluorescence signals were identified as hit compounds.). By the SMM for various hairpins and riboswitches, compounds **5** and **16** (Table 2, **8**, **9**) were identified to which reduced the levels of MALAT1 RNA and then branching morphogenesis in a mammary tumor organoid model. Moreover, because compound **5** has no effect on NEAT1, which has a similar ENE triplex, the compound has some specificity in regulating the downstream gene of MALAT1 and can be confirmed by nuclear magnetic resonance spectroscopy [83]. These findings paved the way for providing new approaches by using molecular probes to treat MALAT1-driven cancers (Fig. 3).

**Table 2:** Small-molecule compounds based on SMM approach

Compound	Name	Target	Structure	Cancer type	References
**7**	Mitoxantrone	Pre-miRNA-21, miR-21		Triple negative breast cancer	[50]
**8**	Compound **5**	LncRNA (MALAT1)		Osteosarcoma, breast cancer	[83]
**9**	Compound **16**				
**10**	Compound **4**	HIV transactivation response (TAR) RNA hairpin		HIV	[86]

Interestingly, this approach has also been used for screening small molecules that selectively target HIV transactivation response (TAR) RNA hairpins. Viral RNA is a type of ncRNA that is widely targeted by small molecules [50]. The first viral RNA regulatory elements that could bind and interfere with by small molecules were TAR and the Rev response element (RRE) [84, 85]. Using an SMM strategy, compound **4** (Table 2, **10**) has been found to bind and stabilize TAR hairpin with a K_d_ of 2.4 μM. The activity of the compound is mediated not by the usual cations, but by hydrophobicity and aromatic substituents on the heterocyclic core, which can be derived from the structure–activity relationship. A combination of biophysical and cellular studies has shown that this chemical type selectively binds to TAR to inhibit HIV-mediated cytopathic disease with minimal toxicity [86].

As a high-throughput technology, small-molecule microarray has received an attention due to their advantages in integration and miniaturization. Although this method has some problems in the aspects of small-molecule fixation, load capacity and sensitivity of signal capture system, it has greatly accelerated the progress of drug discovery, which will be useful to find small-molecule compounds for modulating ncRNAs.

### Structure-based designing approach

The high cost of drug screening hinders the drug development process. However, small-molecule ligands that target known spatial structures can be designed purposefully to overcome this obstacle. Hitherto, these compounds can be grouped into three major groups based on the RNA structures they may target: (1) multiple closely packed helices, (2) irregular and usually bulge-containing secondary structures, and (3) triplet repeats. Briefly, a good RNA target should have enough “information” in the structure [20]. With the more accurate understanding of RNA structure, structure-based designing approach has great potential for application to discovery small-molecule compounds targeting ncRNAs [88]. For example, the first miRNA miR-21 that detected in the human genome has been proved overexpression in human tumors [89, 90]. MiR-21 targeting leucine zipper transcription factor-like 1 (LZTFL1) to promote the proliferation and metastasis in breast cancer cells [91]. Of note, miR-21 expression can be increased in several types of cancers, such as hepatocellular carcinoma, and targets phosphatase, tensin homolog (PTEN) and TP53 [92]. Moreover, miR-21 has been reported to suppress the invasion and metastasis of osteosarcoma [93]. Therefore, it is important to discover more small-molecule inhibitors of miR-21. Because the classical method of miRNA modeling only considers the standard nucleotide face-to-face situation, which cannot solve the non-classical model of hydrogen bond and sugar interaction and nucleotides not in a face-to-face arrangement, this can result in the construction of an imperfect or erroneous model [94]. To solve this problem, the bioinformatics research group led by major proposed a method of miRNA modeling based in principle on MC-Fold [95, 96]. In this approach, a series of nucleotide cyclic motifs (NCM) is used to define the structure of miRNAs, which covers all possible interactions between adjacent nucleotides and will be used for the prediction of the secondary structures of miRNAs.

**Fig. 4:**
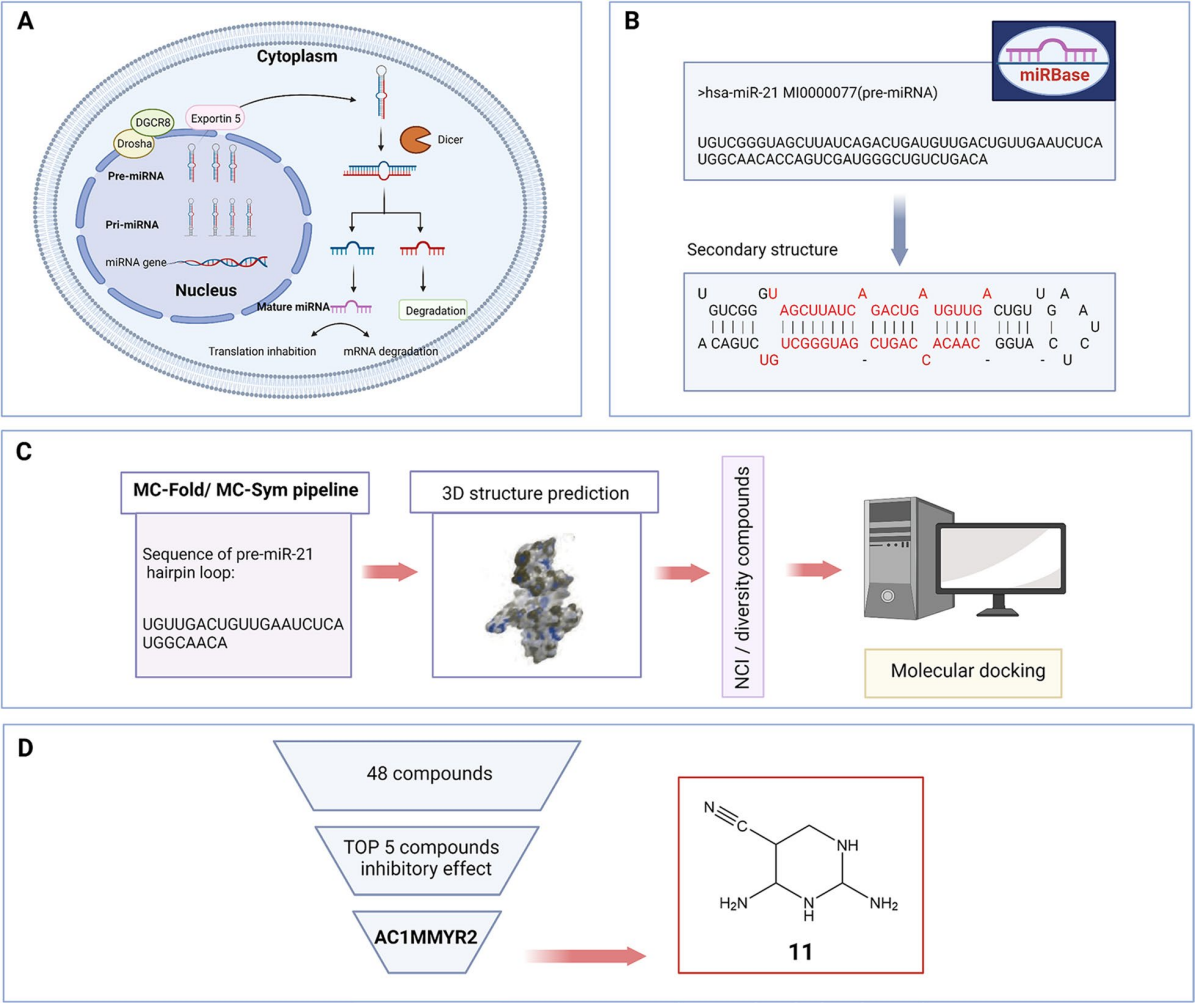
Prediction and screening of compound **11** as a small-molecule compound regulating ncRNA based on the 3D pre-miR-21 model. (**A**) Graphical representation of miRNA production. (**B**) The process of generating sequences of pre-miR-21 hairpin loops (Dicer binding sites on pre-miR-21) from the MIRBASE database. Red bases represented mature miR-21 duplex. (**C**) 3D structure of pre-miR-21 hairpin loop simulated by using the MC-Fold/MC-Sym calculation method. (**D**) After screening the molecular docking results, compound **11** not only has high affinity but also has strong biological activity

With the emergence of the rapid progress of computational methods, remarkable achievements have been made in investigating three-dimensional crystal structure. MC-Fold, MC-Sym and RNA structure prediction programs have successfully predicted several miRNA precursor double helix regions (including Let-7c, miR-19, miR-29a), while the new energy-based method can detect nonclassical base pairs. When using miR-30a, let-7 crystal or NMR structure as reference, the structure initially predicted by MC-Fold and MC-Sym methods can reproduce the basic A-helix of pre-miRNA, and the predicted results for Watson Crick base pairs, swinging base pairs and protruding stem loops are generally consistent with the secondary structure database. The three-dimensional structure of the hairpin ring of pre-miRNA was predicted through the MC-Fold/MC-Sym pipeline, after which high-throughput screening of small molecules that hinder the maturation of miR-21 was performed. Finally, an important small molecule inhibitor of miR-21, AC1MMYR2 (Table 3, **11**), was found, which interacted directly at the binding site of Dicer to prevent pre-miR-21 from cleaving into mature miRNA [87]. It also has been shown to induce PTEN, PDCD4 and Reck to inhibit proliferation, induce apoptosis and inhibit invasion of cancer cells, and reverse epithelial-mesenchymal transition via up-regulating MIR-200a/b and miR-181d. Interestingly, AC1MMYR2 was subsequently found to target the miR-21/CDK5 axis to counter taxol-induced breast cancer metastasis [97]. These results confirm the compatibility and reliability of using MC-Fold and MC-Sym as major miRNA structure prediction tools [98, 99] (Fig. 4).

**Table 3:** Small-molecule compounds based on structural designing approach

Compound	Name	Target	Structure	Cancer type	References
**11**	AC1MMYR2	Pre miR-21, MiR-21		Glioblastomas, breast cancer, colon cancers and gastric cancer, etc.	[87]
**12**	Compound **2**	MiR-17–92 cluster		Polycystic kidney disease, Prostate cancer, and breast cancer	[22]
**13**	AC1NOD4Q	LncRNA (HOTAIR)		Glioblastoma gastric cancer and breast cancer	[23]

The miR-17-92 cluster is regarded as a proto-oncogene, which is highly expressed in many kinds of tumor tissues, such as gastric cancer [100–102]. Serum levels of miR-17-92 cluster members in gastric cancer patients have been reported to be elevated and can be considered as potential biomarkers for early diagnosis of this disease [103]. In another study, the miR-17-92 cluster has been found to play a crucial role in progression and migration of MGC-803 gastric cancer cells, as well as regulating the nuclear factor kappa-B (NF-κB) pathway by directly targeting TRAF3 [104]. Thus, it is necessary to concentrate on designing biogenic compounds that inhibit members of the miR-17-92 cluster [98, 99]. Coincidentally, the three miRNAs belonging to the miR-17-92 cluster (miR-17, -18a, -20a) have the same U bulge (5’G_U/3’CUA) in their Dicer sites. According to this characteristic, compound **2** (Table 3, **12**) that target the degradation of cancer-causing miR-17-92 cluster can be designed and optimized by using a sequence-based method of structure-specific ligand design [22].

Furthermore, small-molecule compounds that regulate lncRNA can also adopt this strategy. HOTAIR was found not only to be involved in breast cancer, gastric cancer, and liver fibrosis, but also in the pathogenesis of glioblastoma, colorectal cancer, and others [105–108]. Importantly, HOTAIR is one of the key lncRNAs that interact with polycomb repressive complex 2 (PRC2), which can promote chromatin remodeling and transcriptional and posttranscriptional regulation [109, 110], making the development of lead compounds inhibiting HOTAIR even more important [111–114]. AC1NOD4Q (Table 3, **13**) is a selectively compound that interferes with the HOTAIR/EZH2 interaction and blocks HOTAIR activity [23]. Briefly, the principle is to use sequence data (212–300 nt) and MC-Fold/MC-Sym programs to construct 3D models. Thus, the three-dimensional hairpin loop structure within the PRC2-binding element of HOTAIR can be predicted, whereby these structures may be targets of small-molecule intervention. Finally, AC1NOD4Q was obtained by screening of binding free energy, solubility, and high affinity. Besides, the biological function of ADQ also has been shown to inhibit the invasion and migration by blocking the β-catenin pathway in MDA-MB-231 cells.

Structure-based design strategies, including data-mining, structure prediction, and computer virtual screening, are a way to accelerate the drug discovery process by using structural information, which can reduce the cost of small-molecule discovery. The technique requires that high-resolution three-dimensional structures of ncRNAs have been obtained, which is not a small difficulty, because the existing techniques has far less understanding of the binding cavities of ncRNAs than proteins. Therefore, structure-based drug design is more suitable for ncRNAs with known structures.

### Phenotype-based screening approach

In addition to the methods mentioned above, phenotypic screening, a method according to the phenotypic changes in a specific environment, is of great significance in discovering RNA-binding small molecules. Intriguingly, traditional drug phenotypic screening is more based on animal disease model screening, by observing the change of the body phenotypic drugs. Linezolid, ribocil, branaplam and SMA-C5 are classic examples of phenotypic screening successes. Taking ribocil (Table 4, **14**) as an example [115], flavin mononucleotide riboswitch is a structural and metabolic reaction factor in the promoter region of the *ribB* gene. FMN riboswitch plays a key role in riboflavin biosynthesis and essential for bacterial growth. Since the first discovery of riboswitch in 2002, it has been recognized as an attractive target [116]. The Merck company used a phenotypic screening method to test a library of about 57,000 small-molecule compounds to identify lead compounds with both antibacterial effects and growth inhibitory properties. The results demonstrate that ribocil can inhibit the production of riboflavin and its metabolites. In summary, the discovery of ribocil strongly supports the notion that phenotypic screening is a good strategy to find binding RNA to small molecules.

**Table 4:** Small-molecule compounds based on phenotype screening and fragment-based approaches

Method	Compound	Name	Target	Structure	Function	References
Phenotype screening	**14**	Ribocil	FMN riboswitch		Antibacterial	[115, 116]
	**15**	Linezolid	tRNA		Antibacterial	[117]
Fragment- based approach	**16**	Hit **5**	Telomeric repeat-containing RNA (TERRA)		Antitumoral	[118]
	**17**	Hit **7**				
	**18**	Hit **8**				
	**19**	Hit **9**				
	**20**	Hit **10**				
	**21**	Hit **11**				

Another classic example of the results of phenotypic screening is Linezolid (Table 4, **15**) an oxazolidinone antibacterial agent, belonging to the broad-spectrum group of agents. It has perfect activity against most of Gram-positive pathogens, including methicillin-resistant Staphylococcus, penicillin-resistant pneumococci, macrolide-resistant Streptococcus and vancomycin-resistant enterococcus [119]. Linezolid binds to a site pocket in the center of ribosomal peptidyl transferase, and overlaps with the amino acyl part of tRNA bound to site A, thereby interfering with the correct location of tRNA [117]. Due to the highly abundance of ribosomes in cells, there is no strict requirement for affinity of Linezolid. Phenotypic screening has been widely used in the drug discovery industry, and while the approach was initially not considered RNA as a potential target, they did find compounds that regulate ncRNAs.

### Fragment-based approach

As the name suggests, the fragment-based method is based on the design and establishment of a compound library composed of fragments of molecules. The bioactivity of the molecules is screened to find candidate fragment molecules; the binding mode and strength of such molecular fragments with the target is then analyzed by nuclear magnetic resonance (NMR), X-ray crystallography, and mass spectrometry (MS), and the lead compounds were obtained by optimizing the structure of the fragments [120, 121]. For example, by applying a method based on ^19^F-NMR fragment screening to identify small-molecule compounds binding to RNA molecules [118]. A library of 355 fluorine-containing compounds was constructed and their interaction with telomere long RNA tested as the target molecule. In the initial screening of 20 molecules, seven compounds were further verified as selective for RNA G-quadruplexes. Hit **5**, Hit **7**, Hit **8**, Hit **9**, Hit **10** and Hit **11** were found to interact with the short Terra structure of two repeat sequences (TERRA2) (Table 4, **16**–**21**). Four compounds showed selectivity for dsDNA and phenylalanine tRNA by ^1^H-NMR and ^19^F-NMR, respectively. When all small-molecule compounds interact with the DNA analogues of TERRA2, they show favorable parallel conformation, which is the main conformation of RNA G-quadruplexes in Terra. These properties make some synthetic binders promising as lead compounds for fragment-based drug discovery. The fragment-based approach has also been used to study the small-molecule compounds that bind to the promoter of the influenza A virus [122].

This is a method to combine moieties, as all compounds are made up of many small fragments. The use of chemical fragments can improve the hit rate of the target by increasing the number of binding possibilities by reducing steric hindrance between them and other positions on the whole target molecule. Once a fragment has been identified, compounds can be made into effective selective inhibitors through a variety of strategies.

### Pharmacological validation approach

Although there are some small-molecule compounds that have been pharmacologically validated, these ncRNA regulating compounds are not created out of thin air. On the contrary, they were generated based on extensive literature research and pharmacological experiments. Bisphenol-A (BPA) (Table 5, **22**) and diethylstilbestrol (DES) (Table 5, **23**) are typical examples. LncRNA HOTAIR was highly expressed in a variety of cancers. Relevant mechanistic studies have shown that HOTAIR plays a key role in the process of proliferation and migration of breast cancer cells, and is transcriptionally regulated by estradiol [123, 124]. Subsequently, it was speculated that exposure to low concentrations of BPA and DES could significantly induce HOTAIR expression in breast cancer cells MCF7 cultured in vitro, and this was subsequently shown to be the case. Since HOTAIR is a lncRNA with trans-transcriptional regulation, this study also demonstrated that BPA and DES can interfere with ncRNA and induce antisense transcription [125].

**Table 5:** Other designing strategies of small-molecule compounds for modulating ncRNAs

Method	Compound	Name	Target	Structure	Cancer type	References
Pharmacological validation	**22**	Bisphenol-A (BPA)	LncRNA (HOTAIR)		Breast cancer	[125]
	**23**	Diethylstilbestrol (DES)				
	**24**	3-(4-amino-1H-benzo[d]imidazole-2-carboxamido)-4-oxo-3,4-dihydroimidazo[5,1-d][1,2,3,5]tetrazine-8-carboxamide	CircRNAs:cirbap2, circCdr1, circZNF292, circHIPK3, circTCF25, LncRNAs: H19, HULC DQ786243, HOXD-AS1, CCAT1		Glioma, Breast cancer	[126]
	**25**	2-O-methylmagnolol	LncRNA GAS5		Skin cancer	[129]
	**26**	Honokiol	MiR-21		Osteosarcoma	[130]
	**27**	Curcumin	MiR-21		Osteosarcoma	[131]

A nitrogen-containing heterocyclic compound that regulates ncRNAs has also been reported. Previous studies have shown that overexpression of alkyl glycerophosphate synthase (AGPs) can increase the growth and migration of many types of cancer cells. Although benzyl isothiocyanate as a natural compound has antitumor activity in many types of cancers, its application is limited due to its toxicity. Thus, 3-(4-amino-1H-benzo[d]imidazole-2-carboxamido)-4-oxo-3,4-dihydroimidazo[5,1-d] [1, 2, 3, 5] tetrazine-8-carboxamide (Table 5, **24**), a new nitrogen-containing heterocyclic compound, was designed and synthesized using computer aided drug design (CADD) technology and AGPs as the target. Finally, the effects of nitrogen heterocyclic compounds have been shown in proliferation and invasion of U251 glioma and MCF-7 breast cancer cells, suggesting that nitrogen-containing heterocyclic compounds can decrease the expression of the circRNAs cirbAP2, cirCZNF292, circhipK3, cirCTCF25, cirCCDR1, as well as known oncogenes, such as lncRNAs DQ786243, HOXD-AS1, H19, CCAT1, and HULC[126].

It is well-known that the active components of many natural products have specific pharmacological activities. In a further study of biological mechanisms, we found that these compounds may play an important role in regulating ncRNAs. For example, magnolol, obtained from the bark of Magnolia officinalis, is a new biphenyl lignin [127]. Methoxylated magnolol seems to possess enhanced anti-inflammatory activity. 2-o-methylmagnolol (Table 5, **25**) was found to up-regulate the lncRNA growth arrest specific 5 (GAS5), promote the apoptosis of skin cancer cells, and improve anti-tumor activity against these cells [128, 129]. Honokiol (Table 5, **26**), an isomer of magnolol, has antitumor effects on various types of cancer. Honokiol have recently been confirmed to induce the abnormal expression of miRNAs in human osteosarcoma cells, with miR-21 being one of the most significantly down-regulated miRNAs. The specific mechanism is that honokiol inhibits the proliferation of osteosarcoma cells and induces their apoptosis of osteosarcoma cells by regulating miR-21/PTEN/PI3K/Akt pathways [130]. Coincidentally, based on pharmacological validation, curcumin (Table 5, **27**) has been reported to reduce miR-21 expression and to have anti-osteosarcoma activity [131]. It is undeniable that pharmacological verification strategies can bring us small-molecule compounds that regulate ncRNAs, but their specific binding sites and regulatory modes should be further explored.

### Other designing strategies

There are several other strategies for seeking compounds that can regulate ncRNAs, including bioinformatics approaches, one-bead-two-compound (OBTC) screening and microscale thermophoresis [132–134]. The prediction of candidate miRNA-targeted drugs is performed by calculating the transcriptional similarity of small molecules and miRNAs [135]. First, 6100 sets of microarray data of small interfering molecules were obtained from connectivity map databases, involving 1309 small-molecule compounds. They then conducted a comprehensive scan of the Gene Expression Omnibus database, obtaining 124 sets of gene expression profiles of miRNA transfections, and finally screened out 39 sets of microarray data, involving 25 miRNAs. The Sam method or fold-change was used to identify the differential expression of probes before and after small-molecule interference and miRNA transfection, and they were then used as the transcription reaction of small molecule and miRNA. Finally, the enrichment score and statistical significance of each pair of small molecules and miRNAs was calculated, and the small molecules with negative scores were tested as candidate drugs to reverse the function of the miRNA. Taking results at the significance level of *P* < 0.01, a total of 1937 pairs of significant associations was identified among the 1309 small molecules and 25 miRNAs, of which 415 were FDA approved, involving 859 pairs. Next, they evaluated the efficiency of the method by using the small molecules that did affect the expression of miRNAs. The *P* value of the hypergeometric distribution test was 6.81 × 10^–11^, indicating that the predicted results significantly identify the relationship between drugs and miRNAs. Identifying the relationships between drugs and miRNAs from the perspective of transcriptional similarity provides a new clue on the development of miRNA-targeted drugs.

## Concluding remarks and future perspectives

Currently, the strategy of regulating RNA, especially ncRNAs, gradually constitutes a widely recognized trend for drug design and development. To our knowledge, ncRNAs are extremely abundant in the human genome, accounting for about 70%, one order of magnitude higher than protein coding sequences. Besides, the spatial configuration of such biomolecules contains sufficient information to be directly targeted. Therefore, designing strategies to use small-molecule compounds for regulating ncRNAs would greatly broaden the range of druggable targets, which will be a beneficial supplement to the mainstream strategies targeting proteins. On the other hand, accumulating evidence has recently shown that ncRNAs regulate gene expressions in many ways, including the transcriptional, post-transcriptional and even epigenetic levels, and are involved cell proliferation, apoptosis, and cell cycle control during carcinogenesis. Importantly, the biological association between ncRNA and cancer suggests that ncRNA may be a promising druggable target for cancer drug discovery. Collectively, the development of small-molecule drugs that regulate ncRNAs has become an exciting field.

Inevitably, the emergence of new fields will always face many challenges and limitations. For instance, unknown and dynamic three-dimensional conformation of ncRNAs may cause some limitations on designing small-molecule drugs based on binding pockets. In fact, the idea that RNA is “flexible” is not entirely true, because some RNA parts are so rich in structure, drug design can be easily achieved if the region is precisely located. Based on RNA chemical probe technology, the Selective 2'-hydroxyl acylation analyzed by primer extension (SHAPE) tool was designed to help identify RNA secondary structure motifs [136]. The leader of RNA-targeting drugs Disney laboratory have constructed a database, Inforna, based on two-dimensional combinatorial screening, and found several small molecules possessing activity by target RNAs in both in vitro and in vivo disease models. Inspiringly, using the Inforna technique, Targaprimir-96 was found to activate RNase L and inhibit miR-96 in triple negative breast cancer cells, which resulted in increased expression of the *FOXO1*, thus triggering cancer cell death [137]. Moreover, the dynamic set of RNA three-dimensional structures can be obtained by NMR [138].

**Fig. 5:**
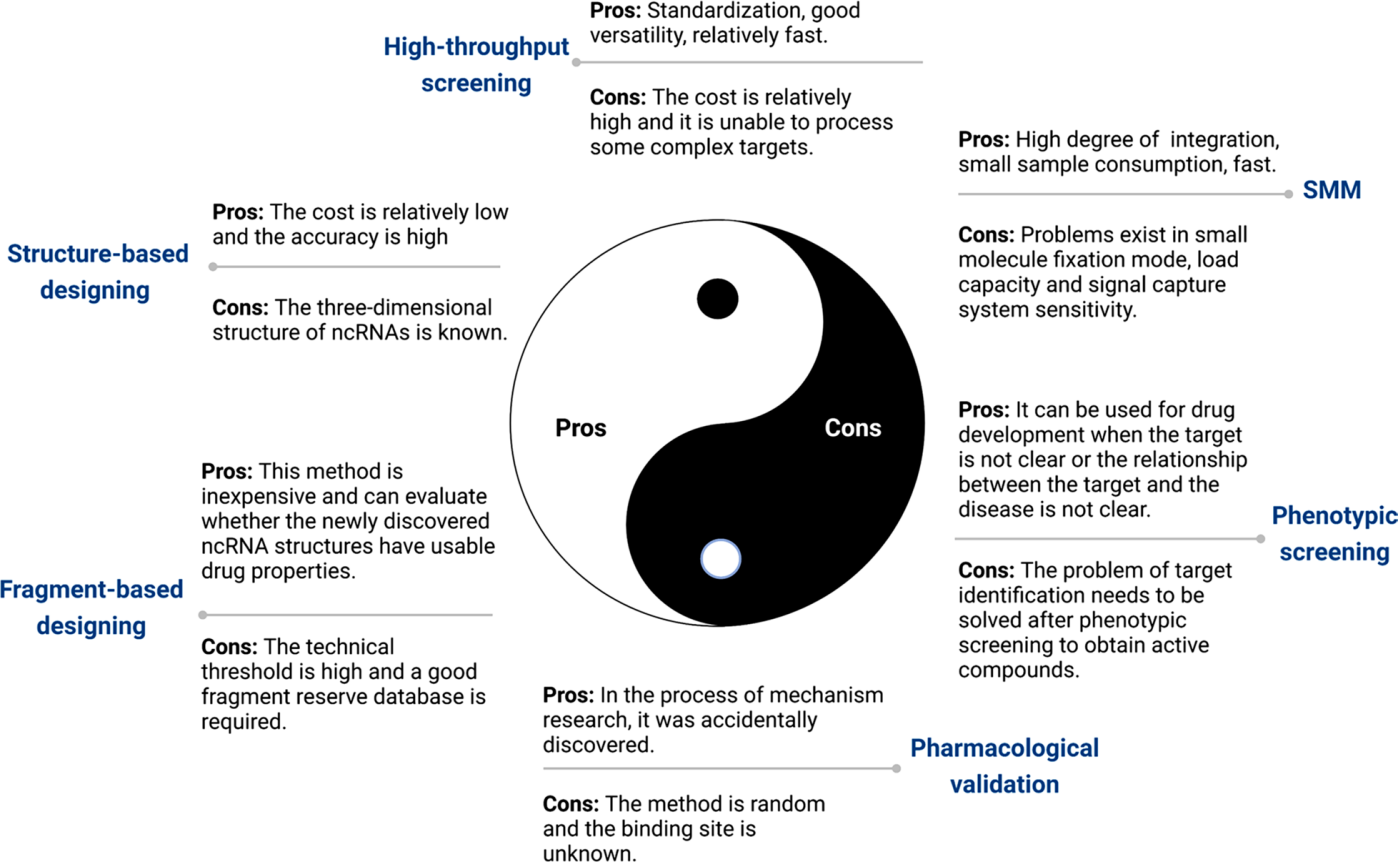
Pros and cons of designing strategies of small-molecule compounds for modulating ncRNAs in cancer

Robicil, Branaplam and other small-molecule compounds regulate the RNA of bacteria and viruses, which makes small-molecule compounds for modulating ncRNA have special characteristics. However, other compounds have been demonstrated to modulate ncRNAs, including AC1MMYR2, AC1NOD4Q, Enoxacin, Honokiol, and even MRG-106, a small-molecule inhibitor of miR-155 for clinical trials. More interestingly, these compounds also have potential therapeutic applications in other non-oncology diseases, such as HIV, HCV, and antimicrobial diseases. Therefore, we summarize the pros and cons of designing strategies of small-molecule compounds for modulating ncRNAs: (1) High-throughput screening approach, (2) Small-molecule microarray approach, (3) Structure-based designing approach, (4) Phenotype-based screening approach, (5) Fragment-based approach, (6) Pharmacological validation methods (Fig. 5). Small-molecule microarray methods (e.g., AbsorbArray) and high-throughput screening methods can save time and rapidly focus on a reduced target range. Three-dimensional models of ncRNAs can be constructed by using MC-Fold and MC-Sym programs, which can improve the accuracy.

Now, we are at the beginning of efforts to decipher small-molecule compounds that effectively regulate different types of ncRNAs. Compounds with endogenic protein targets need to be considered for their potential off-target action or side effects. In vitro*,* the mechanism of action of small molecules with pharmacological activity and the specificity of compounds remain to be discovered. Fortunately, applications of multi-omics (transcriptomics, proteomics etc.) may distinguish the disease-related ncRNAs and protein targets, as well as their abundance in pathological tissues. For different elements of RNA (A, U, G, and C, four primary nucleotide monomeric units) and protein (22 proteinogenic amino acids) result highly structured RNAs differ from proteins in physicochemical properties and accessibility that allow utilization structural optimization to improve the selectivity of small-molecule compounds toward an objective RNA target. With the development of cryo-electron microscopy, deep machine learning technology and artificial intelligence (AI), the RNA structure and the number of small molecules that can selectively target and modulate ncRNA will be increased dramatically. In a nutshell, we hope more small-molecule drugs for regulating ncRNAs will be discovered by using the above-mentioned designing strategies, which will not only contribute to a breakthrough in small-molecule drug discovery, but provide a new opportunity for cancer therapy.

## Data Availability

Not applicable.

## Abbreviations

AGPs: Alkyl glycerophosphate synthase
ALIS: Automatic ligand recognition system
AS-MS: Affinity selection mass spectrometry
ASO: Antisense oligonucleotide
BLACAT1: Bladder cancer-associated transcript 1
BPA: Bisphenol-A
CADD: Computer-aided drug design
CDC42: Cell division cycle 42
ceRNA: Competitive endogenous RNA
circRNA: Circular RNA
DDX 5: DEAD-box RNA helicase 5
DES: Diethylstilbestrol
ENE: Element for nuclear expression
ESI: Electrospray ionization
FT-ICR: Fourier transform ion cyclotron resonance
GAS5: Growth arrest specific 5
HER 2: Human epithelial growth factor receptor 2
HiT-StARTS: High-throughput structure activity relationship deconvolution through sequencing
HOTAIR: HOX transcript antisense intergenic RNA
lncRNA: Long non-coding RNA
LZTFL1: Leucine zipper transcription factor-like 1
MALAT1: Metastasis-associated lung adenocarcinoma transcript 1
MALDI: Matrix-assisted laser desorption ionization
MIAT: Myocardial infarction-associated transcript
miRNA: MicroRNA
mRNA: Messenger RNA
MS: Mass spectrometry
NCM: Nucleotide cyclic motifs
ncRNAs: Non-coding RNAs
NF-κB: Nuclear factor kappa-B
NIH-CC: NIH clinical collection
OBTC: One-bead-two-compound
PTEN: Phosphatase and tensin homolog
PDCD4: Programmed cell death 4
pre-miRNA: MiRNA precursor
pri-miRNA: Primary miRNA
ROCK1: Rho-associated coiled-coil containing protein kinase 1
RRE: Rev response element
rRNA: Ribosomal RNA
siRNA: Small interfering RNA
SMM: Small-molecule microarray
STAT3: Signal transduction and activator of transcription 3
TAR: HIV transactivation response
TARID: TCF21 antisense RNA inducing demethylation
tiRNAs: TRNA half fragments
TRF: TRNA-derived fragments
tRNA: Transfer RNA
